# A test of the goodness of fit of the generic metacognitive model of psychopathology symptoms

**DOI:** 10.1186/s12888-019-2266-5

**Published:** 2019-09-18

**Authors:** Henrik Nordahl, Ingunn Harsvik Ødegaard, Odin Hjemdal, Adrian Wells

**Affiliations:** 1 Department of Psychology, Norwegian University of Science and Technology, Trondheim, Norway; 2 St. Olavs Hospital, Division of Psychiatry, Trondheim University Hospital, Trondheim, Norway; 3 School of Psychological Sciences, Faculty of Biology, Medicine and Health, University of Manchester, Manchester, UK; 4 Greater Manchester Mental Health NHS Foundation Trust, Prestwich, UK

**Keywords:** Metacognitive beliefs, Attentional control, S-REF model, Generic metacognitive model, Depression, Generalized anxiety, Social anxiety

## Abstract

**Background:**

Common mental disorders such as depression and anxiety frequently co-occur and may share etiological mechanisms. The metacognitive model is based on the principle that there are common pathological mechanisms across disorders that account for comorbidity and therefore can be conceptualized in one generic model. A central prediction of the model is that particular metacognitive beliefs concerning the value of worry, and the uncontrollability and danger of cognition are positively correlated with psychopathology symptoms. In the present study, we set out to test the overall fit of this model by assessing generic metacognitive beliefs and judgements of attention control capacity as predictors of common and frequently co-occurring emotional distress symptoms.

**Methods:**

In a cross-sectional design, 645 participants gathered at convenience completed a battery of self-report questionnaires.

**Results:**

Structural equation modelling indicated a good model fit for the generic metacognitive model, and the predictors accounted for 93% of the variance in distress consisting of depression-, generalized- and social anxiety symptoms.

**Conclusions:**

This finding supports the generic model and the implication that it can be used as a basis to formulate and treat multiple presenting problems.

## Background

Evidence-based guidelines for mental disorders, such as those provided by the National Institute for Health and Care Excellence (NICE), are based on official classification systems that assume psychopathology can be defined as categorical constructs that are distinct and independent from other constructs. This has led to a collection of specific treatments for specific disorders. For example, specific and distinct variants of cognitive-behavioural therapy (CBT) are recommended as the treatment of choice for major depressive disorder [1] generalized anxiety disorder [2] and social anxiety disorder [3].

The validity of psychopathology categories has been extensively criticized [4] and it has been suggested based on the literature on information processing styles in anxiety and depression that similarities rather than differences should be emphasized in conceptualization and treatment of common mental disorders [5]. More recently, the idea of common factors is supported by research that shows high rates of comorbidity [6], that a substantial proportion of patients do not respond to the recommended disorder-specific treatments [7–9], and that specific mental disorders seem to be manifestations of broader underlying trait dimensions [10–12]. A transdiagnostic approach to conceptualization of psychopathology and psychological vulnerability has therefore been suggested as a potential solution.

One perspective that has advanced our understanding of transdiagnostic factors implicated in emotional disorders is the Self-Regulatory Executive Function (S-REF) model [5, 13] also known as the metacognitive model of psychological disorders [14]. This model grew out of dissatisfaction with how psychotherapeutic models did not capture important common aspects of maladaptive thinking found in most disorders and the factors that might control it [5].

The metacognitive model identified a general negative style of thinking across psychological disorders, called the *Cognitive Attentional Syndrome* [5]. The CAS is comprised of perseverative thinking, predominantly worry and rumination, attentional monitoring of perceived threats, and maladaptive coping strategies. The CAS interferes with self-regulation by impairing emotional processing, locking cognition onto perceived threats, strengthening negative appraisals and beliefs, and preventing corrective learning experiences. Furthermore, the model proposes that the CAS is directed by an underlying meta-level of cognition, which includes explicit knowledge about thinking, memory and attention (i.e. metacognitive beliefs), and implicit non-verbal rules or programs that guide thinking [14]. Thus, emotional disorder symptoms may be the output of running a particular metacognitive plan that is followed by the activation of its corresponding CAS strategies. For example, the belief that worrying is uncontrollable (e.g., “When I start worrying I cannot stop”) may be accompanied by reduced effort to inhibit worrying and monitoring and avoidance of worry triggers. Moreover, the belief that one has low attentional control capabilities may lead to maintenance of distress because it prohibits the person from disengaging the CAS [5].

Together with maladaptive metacognitive beliefs, the S-REF model emphasizes executive functions and their role in top-down regulation of cognitive style. One of these components is attentional control which may facilitate the ability to disengage from perseverative self-attention and the CAS [5, 13]. Thus, individual differences in attention control capacities (in the form of metacognitive beliefs about control or as individual differences in executive functions) could play a role in symptoms and disorders.

The metacognitive approach is based on the principle that there are core similarities in underlying mechanisms across symptoms and disorders. This is not to say that the metacognitive model does not recognize that there can be content specificity at the metacognitive and cognitive levels, but some of the similarities between disorders are seen as more important than the differences. Especially negative metacognitive beliefs about the control of cognition, which is given central importance [14]. The aim of the present study was therefore to examine the model fit of this generic metacognitive model. We chose to model depression-, generalized- and social anxiety symptoms as a latent distress factor as they are prevalent and often highly comorbid [6, 15, 16], and because the World Health Organization has ranked depression as the single largest, and anxiety disorders (of which social- and generalized anxiety are amongst the most prevalent) as the sixth largest contributor to global disability [17].

To specify the metacognitive variables in the generic metacognitive model, we used the gold-standard Metacognitions Questionnaire-30 (MCQ-30;[18]) which assesses metacognitive knowledge in the form of positive beliefs about worry, beliefs about the uncontrollability and danger of worry, judgements of confidence in memory, beliefs about the need to control thoughts, and cognitive self-consciousness. These belief domains have been associated with symptoms of depression, generalized anxiety (see [19] for a review) and social anxiety [20, 21]. Previous studies have shown that metacognitive beliefs are prospective predictors of anxiety and depression symptoms [22–24].

In addition to the MCQ-30, we also included the Attentional Control Scale (ACS; [25]). The ACS was originally developed to assess core individual differences in attention control capacity (i.e. executive functioning), but more recent studies indicate that it more likely assesses subjective judgements of attentional control capacities (i.e. metacognitive beliefs) rather than actual cognitive ability (e.g., [26, 27]). We therefore assume that the ACS offers a measure of metacognitive beliefs about attention even though there is some overlap between the ACS and behavioural assessment of attention control abilities (e.g., [28]). ACS scores have been identified as negatively related to depression [29], generalized anxiety [30], and social anxiety [31].

Previous studies have reported that both generic metacognitive beliefs and attentional control factors as assessed with the ACS explain unique variance in test-anxiety [32], state anxiety in students before end-of-year examinations [33], decisional procrastination [34], and anxiety in children with and without anxiety disorders [35]. However, to the authors’ knowledge, no previous studies have evaluated the structural relationships between generic metacognitive beliefs and attentional control beliefs as underlying factors of common and frequently comorbid distress symptoms. Our hypotheses were as follows; 1) generic metacognitive beliefs will be significantly and positively correlated with all types of symptoms; 2) self-report attentional control will be significantly and negatively correlated with generic metacognitive beliefs and all types of symptoms; 3) the hypothesized generic metacognitive model of emotional distress will show an acceptable fit to the data; and 4) the metacognitive factors will account for substantial variance in the latent symptom variable.

## Methods

### Participants and procedure

Participants were invited to an online survey on psychological factors in emotional distress through social media (Facebook). The study was conducted in Norway, where ethics approval is not required for surveys that collect non-identifying data. However, the study was registered with the Norwegian Centre for Research Data (NSD; ref. nr. 59,447) in line with local legislation as we used “SelectSurvey”, a survey software provided by the faculty at the first authors’ university, to collect the data. Several Norwegian voluntary mental health organizations helped spread information about the survey to their social media followers. Except being 18 years or above, there were no exclusion criteria. The first page of the survey constituted an information sheet that provided the necessary information about the study and its purpose, and stated that moving to the next page would be regarded as consent to participate.

A total of 645 participants completed the survey. In the total sample, the mean age was 36.26 (*SD* = 14.11) and 533 (82.6%) of the participants were female. As for marital status, 216 (33.5%) reported to be single, 69 (10.7%) reported to be in a relationship, 314 (48.7%) reported to cohabit or to be married, 39 (6.0%) reported to be separated or divorced, 5 (0.8%) reported to be widowed, while two (0.3%) did not report their marital status. In terms of occupational status, 195 (30.2%) reported to be students, 279 (43.3%) reported to be working, 10 (1.6%) reported to be unemployed, 14 (2.2%) reported to be on short-term sick leave, 130 (20.2%) reported to be on long-term sick leave (> 1 year), and 17 (2.6%) reported to be retired. In addition, 255 (39.5%) reported to have a higher education (completed 3 years or more at a university or equivalent).

### Measures

The Patient Health Questionnaire (PHQ-9; [36]) is a 9-item self-report questionnaire based on DSM-IV criteria assessing severity of depression symptoms. The respondent is asked to indicate how often during the past 2 weeks one has experienced symptoms of depression (e.g. “Little interest or pleasure in doing things”, “Feeling tired or having little energy”), along a 4-point scale, ranging from 0 (“not at all”) to 3 (“nearly every day”). The PHQ-9 has shown good internal consistency (*α* = .89) [37]. In the current study the internal consistency was excellent (*α* = .93).

Generalized Anxiety Disorder Scale (GAD-7; [38]) is a 7-item self-report questionnaire assessing severity of generalized anxiety disorder symptoms. As in the PHQ-9, respondents are asked to indicate how often during the past 2 weeks one has experienced symptoms of anxiety such as “Becoming easily annoyed or irritable” and “Being so restless that it is hard to sit still”. Each item is scored on a scale ranging from 0 (“not at all”) to 3 (“nearly every day”). The instrument has shown excellent internal consistency (*α* = .92) [38]. In the current study the internal consistency was excellent (*α* = .93).

Fear of Negative Evaluation (FNE; [39]) is a 30-item self-report questionnaire assessing severity of social anxiety symptoms. Respondents are asked to rate 30 statements (e.g. “Sometimes I think I am too concerned with what other people think of me”, “I am afraid that people will find fault with me”) as true or false. A total score can be derived by summarizing positive responses to items characterizing social phobia (range 0 to 30), and a higher total score indicate greater levels of social fears and anxiety. The instrument has demonstrated excellent internal consistency (*α* = .94; [39]), which is also was in the current study (*α* = .95).

Metacognitions Questionnaire 30 (MCQ-30; [18]) is a 30-item self-report measure assessing metacognitive beliefs (beliefs about cognition). The scale has five subscales; a) positive beliefs about worry (pos; e.g. “I need to worry in order to work well”), b) negative beliefs about uncontrollability and danger of worry (neg; e.g. “When I start worrying I cannot stop”), c) cognitive confidence (cc; e.g. “I have a poor memory”), d) beliefs about the need to control thoughts (nc; e.g. “It is bad to think certain thoughts”), and e) cognitive self-consciousness (csc; e.g. “I monitor my thoughts”). Each item is rated on a 4-point scale ranging from 1 to 4 (1 = do not agree; 2 = agree slightly; 3 = agree moderately; 4 = agree very much) and reflect how much the respondent agrees with the statement. The MCQ-30 subscales have shown acceptable to good internal consistency (*α* = .77–.89) [22, 40], and in the current study the internal consistency was good (pos: *α* = .84, neg; *α* = .90, cc; *α* = .89, nc; *α* = .85, csc; *α* = .79).

Attentional Control Scale (ACS; [25]) is a 20-item self-report questionnaire intended to assess individual differences in attentional control capacity. More recent psychometric evaluation has indicated a two-factor solution (e.g., [41, 42]) consisting of; a) attention focusing (e.g. “It’s very hard for me to concentrate on a difficult task when there are noises around”) and b) attention shifting (e.g. “I have a hard time coming up with new ideas quickly”). Out of the original items, these factors consist of seven (item number 1, 2, 3, 6, 7, 8, 12) and five items (10, 13, 17, 18, 19) respectively. Each item is rated on a 4-point scale ranging from 1 (“almost never”) to 4 (“always”). A higher total score indicates better judgements of attentional control abilities. The ACS focusing subscale has shown good internal consistency (*α* = .82), and the shifting subscale acceptable internal consistency (*α* = .71) [42]. In the current study, the internal consistency was good for the focusing (*α* = .88) and for the shifting subscale (*α* = .76).

### Overview of statistical analyses

**Fig. 1:**
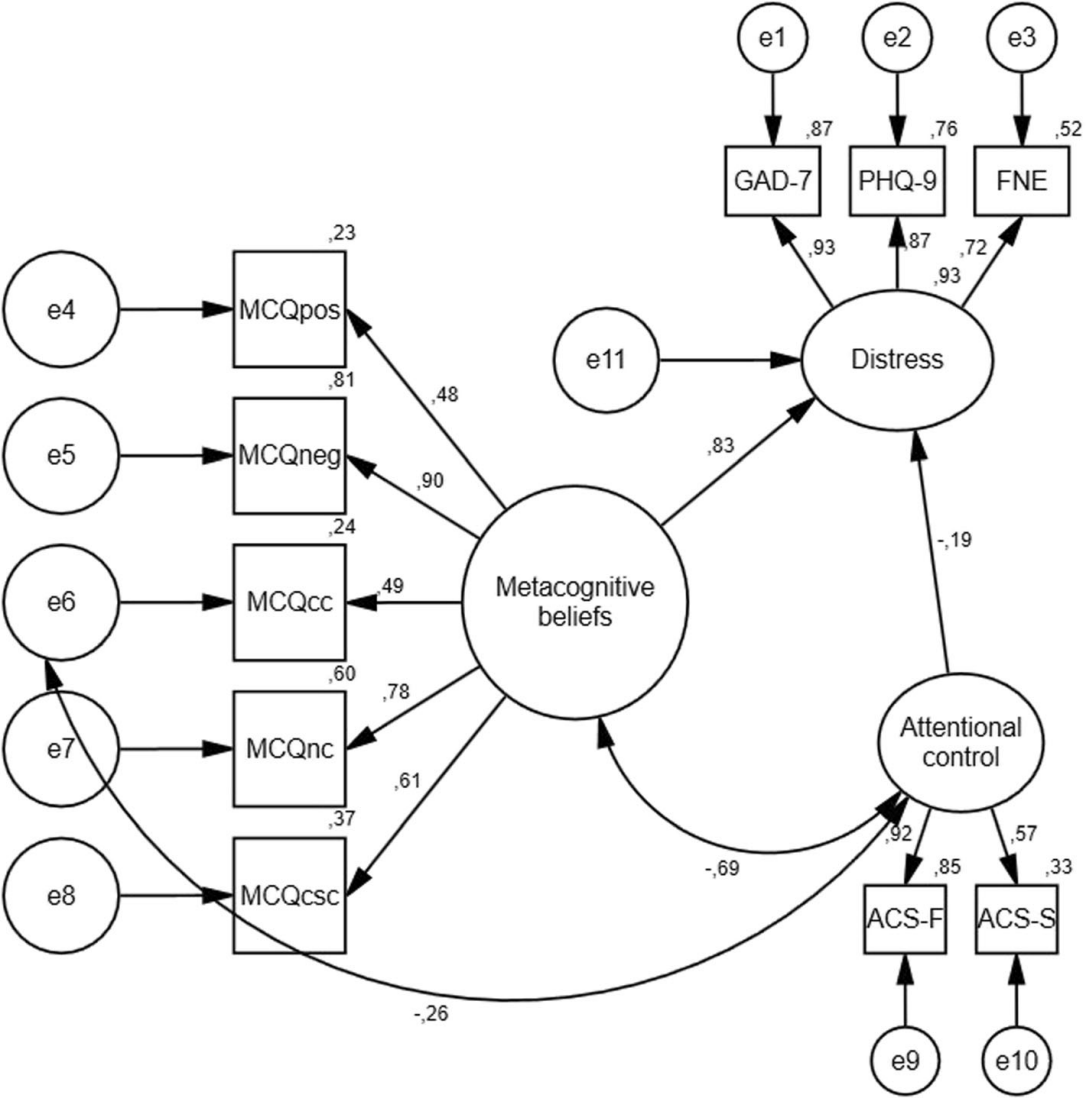
Testing the generic metacognitive model. Structural equation model of the relationship between generic metacognitive beliefs, metacognitive beliefs about attentional control and emotional distress (*N* = 645). Ellipses represent latent variables, and rectangles represent observed variables (indicators). PHQ-9 = Patient Health Questionnaire, GAD-7 = Generalised Anxiety Disorder Scale, FNE = Fear of Negative Evaluation, MCQpos = positive metacognitive beliefs, MCQneg = negative metacognitive beliefs, MCQcc = cognitive confidence, MCQnc = need for control, MCQcsc = cognitive self-consciousness, ACS-F = attentional control focusing, ACS-S = attentional control shifting. The figure shows standardized coefficients (all significant at the .01 level)

All analyses were conducted using IBM SPSS Statistics version 25 and IBM SPSS Amos 25 Graphics. Bivariate correlations were used to explore the basic associations between the variables. Structural equation modelling (SEM) was employed to evaluate the fit of an overall model where a latent metacognition factor (consisting of the MCQ-30 subscales) and a latent attentional control factor (consisting of the ACS subscales) were used. A latent psychological distress/symptom factor (which consisted of the variables PHQ-9 (depression), GAD-7 (generalized anxiety) and FNE (social anxiety)) was specified. We tested the fit of the model (Fig. 1) in which the three latent factors (metacognitions; MCQ-30), attentional control, and transdiagnostic symptoms were correlated as shown. Modification indices were used to identify potential local misfits and secondary loadings were added if identified and consistent with metacognitive theory. Four commonly-recommended fit statistics were used to evaluate the models [43, 44] the comparative fit index (CFI), the Tucker Lewis index (TLI), the standardized root mean square residual (SRMR) and root mean square error of approximation (RMSEA). The CFI and TLI should be above .95, the SRMR should be less than .08, and the RMSEA should be below or close to .06 and the upper limit of the 90% RMSEA confidence interval should not exceed .10 to represent a good fit.

## Results

### Correlational analyses

Table 1 presents descriptive statistics and bivariate correlations between all variables. All of the correlations were significant at the .01 level. Distress domains were positively inter-correlated and were positively correlated with metacognitive belief domains and negatively correlated with attentional control. Moreover, metacognitive belief domains and attentional control were negatively correlated with each other. That is to say greater maladaptive metacognitions were associated with poorer attention control. Among the metacognitive belief domains, negative beliefs concerning uncontrollability and danger consistently showed the strongest association with symptoms, followed by need for control. Out of the attentional control domains, focusing consistently showed the strongest association with symptoms, and was more closely related to metacognitive beliefs domains than shifting.

**Table 1:** Mean values and standard deviations for all variables and bivariate inter-correlations (*N* = 645)

	2.	3.	4.	5.	6.	7.	8.	9.	10.	Mean (*SD*)
1. PHQ-9	.818*	.617*	.359*	.738*	.504*	.659*	.435*	−.632*	−.437*	10.08 (7.29)
2. GAD-7		.662*	.432*	.816*	.435*	.683*	.527*	−.633*	−.387*	7.77 (5.94)
3. FNE			.345*	.640*	.371*	.549*	.362*	−.517*	−.404*	16.88 (9.30)
4. MCQpos				.376*	.228*	.462*	.383*	−.285*	−.148*	9.06 (3.26)
5. MCQneg					.445*	.679*	.568*	−.592*	−.348*	12.51 (5.06)
6. MCQcc						.387*	.246*	−.527*	−.289*	11.53 (4.92)
7. MCQnc							.510*	−.504*	−.277*	10.15 (4.32)
8. MCQcsc								−.326*	−.113*	12.84 (4.14)
9. ACSfoc									.526*	18.97 (4.88)
10. ACSshi										12.12 (3.14)

*SD* standard deviation, *PHQ-9* Patient Health Questionnaire, *GAD-7* Generalised Anxiety Disorder Scale, *FNE* Fear of Negative Evaluation, *MCQpos* positive metacognitive beliefs, *MCQneg* negative metacognitive beliefs, *MCQcc* cognitive confidence, *MCQnc* need for control, *MCQcsc* cognitive self-consciousness, *ACSfoc* attentional control focusing, *ACSshi* attentional control shifting

*p < .01

### Structural equation modelling

Structural equation modelling was conducted to test the metacognitive model and evaluate relationships between generic metacognitive beliefs, attentional control, and emotional distress. Before taking potential secondary loadings into consideration, the basic model showed the following fit indices: χ^2^(32) = 220.008, *p* < .01, CFI = .95, TLI = .93, SRMR = .05, RMSEA = .10 (90% CI = .08–.11), indicating mixed results concerning model fit. Modification indices indicated a secondary loading that was theoretically consistent, between the observed variable MCQ-30 cognitive confidence and the latent variable attentional control which we included in the final model. The suggested structural equation model is presented in Fig. 1 and showed the following fit indices: χ^2^(31) = 158.771, *p* < .01, CFI = .97, TLI = .95, SRMR = .04, RMSEA = .08 (90% CI = .07–.09), indicating a good model fit to the data. The specified predictors explained 93% of the variance in the psychological distress latent variable, identified by the squared multiple correlation (adjusted for number of used indices). Moreover, further inspection of the standardized coefficients in the model showed that negative beliefs about the uncontrollability and danger of worry made the strongest contribution to the latent generic metacognitive beliefs variable, while attention focusing made the strongest contribution to the latent attentional control variable. Furthermore, the latent generic metacognitive beliefs variable made a stronger contribution to the latent symptom variable than the latent attentional control variable.

## Discussion

The primary aim of the current study was to evaluate the fit and hence validity of the generic metacognitive model. In order to do this, we used structural equation modelling where we specified two latent metacognitive factors consisting of metacognitive beliefs and beliefs about attentional control as predictors of a third latent distress factor comprised of depression-, generalized anxiety- and social anxiety symptoms.

In line with our hypotheses, all domains of metacognitive beliefs and attentional control were significantly correlated with symptoms in the expected direction, and the strengths of correlations was moderate to strong. These findings indicated that greater endorsements of maladaptive metacognitive beliefs about thoughts and beliefs in low attention control capabilities were associated with higher distress levels. Furthermore, metacognitive belief domains showed a small to moderate negative correlation with attentional control subscales, indicating that greater endorsements of metacognitive beliefs concerning worry are associated with beliefs of lower attentional control capabilities.

Negative metacognitive beliefs about the uncontrollability and danger of worry followed by need for control of thoughts showed the strongest association with symptoms among the metacognitive belief domains. Furthermore, attention focusing consistently showed a stronger association to symptoms and to metacognitive belief domains than attention shifting.

Structural equation modelling was conducted to test the fit of the suggested generic metacognitive model and evaluate relationships between the specified latent variables (metacognitive beliefs, attentional control, and symptoms). This basic model included no secondary loadings and indicated mixed results concerning model fit. Following examination of modification indices, a covariation between the error term of the observed variable MCQ-30 cognitive confidence and the latent variable attentional control was included in the final model as it was assessed to be theoretically consistent. Specifically, the original Metacognitions Questionnaire 65-item version [45] assessed confidence in attention as part of its cognitive confidence factor, but these items were not included in the shortened version (MCQ-30; [18]). The suggested covariate likely represents the fact that cognitive confidence is a marker of confidence in memory as well as other cognitive capacities such as attention, which in the present model is better accounted for by the latent attentional control factor.

The final model indicated an acceptable to good overall model fit to the data. The chi-square was found to be significant, which indicates a poor fitting model, but the chi-square statistic is very sensitive to sample sizes and models with larger numbers of observed variables [46]. However, the CFI, TLI and SRMR indicated a good fitting model, whilst the RMSEA indicated an acceptable fit. In line with the bivariate correlations, the latent metacognitive beliefs factor was significantly and negatively associated with the latent attentional control factor. Both the generic metacognitive factor and the attention control factor explained independent variance in the distress factor, and together accounted for 93% of the variance. The generic metacognitive factor made a stronger contribution to the latent symptom variable compared to the attentional control factor, suggesting that generic metacognitive beliefs might be the most important target for treatment interventions. Moreover, negative metacognitive beliefs about the uncontrollability and danger of worry was the strongest contributor to the latent generic metacognitive beliefs variable, suggesting that this belief domain may be particularly important to modify in treatment. Notably, attention focusing contributed more to the latent attention factor than attention switching perhaps indicating a greater relative importance.

The structural equation model is regarded as a stringent test of models, but one must also keep in mind that several models consisting of the same latent variables and number of relationships between them could provide equally good fit to the data. However, models tested with SEM should be specified by theory in advance, and the most important question to be answered here is; does the model specified by the theory fit? Since this is specified a-priori, there is of course the possibility that the fit will be poor.

The present findings are in line with the metacognitive model of psychological disorders [5, 14] and with previous studies that have confirmed a role for metacognitive beliefs [19, 20] and attentional control [29–31] in psychopathology. Moreover, previous studies have found support for an association between greater maladaptive metacognitive beliefs and lower attentional control, and that these constructs can explain individual variance in several types of psychopathology [32–35, 47]. One previous study reported that attentional control moderated the relationship between the CAS (including metacognitive strategies and beliefs as a total score) and depression- and anxiety symptoms [48]. Previous studies have also indicated that the same metacognitive factors are implicated in different types of distress and that they may explain comorbidity. For example, negative metacognitive beliefs and cognitive confidence correlate with both depression [49] and social anxiety [50], and with depression symptoms in patients with social anxiety disorder [51]. However, the present study expands on previous studies by identifying the structural relationships between the central elements in the generic metacognitive model and by demonstrating the statistical fit of a generic metacognitive formulation of common and frequently co-occurring symptoms.

The present study supports the data from clinical practice. For example, metacognitive therapy (MCT) for major depressive disorder is not only an effective treatment for depressive symptoms, but also has an impact on anxiety and work ability [52], neuropsychological functioning [53], comorbid diagnoses [54], and interpersonal problems [55]. In addition, several studies have evaluated the utility and efficacy of the generic form of MCT: transdiagnostic MCT was more beneficial than treatment as usual in a controlled trial performed in a natural clinical setting in a heterogeneous patient sample [56]. In a randomized feasibility trial, transdiagnostic group MCT was more beneficial than transdiagnostic mindfulness based stress reduction (MBSR) in a mixed disorder sample [57]. In a randomized controlled trial comparing the efficacy of disorder-specific CBT and transdiagnostic MCT for patients with complex and comorbid anxiety disorders, MCT was associated with better outcomes pre to post treatment [58].

There are a number of limitations to the present study that must be acknowledged. The sample was gathered at convenience using online sampling and consisted of substantially more females than males that may compromise the generalizability of the findings. Moreover, we employed a cross-sectional design, and there may therefore be other models that fit the data equally well. Thus, no causal inferences can be made based upon these data. In addition and as noted in the introduction, the ACS may be a better measure of subjective judgements of attentional control capacities (i.e. metacognitive beliefs) rather than actual cognitive ability. Future research should use longitudinal designs and have more control over the samples used. There is also a need for further evaluation of the generic metacognitive model using objective measures of executive functioning such as attentional control. The fit and relevance of the generic metacognitive model should also be tested in clinical samples. Moreover, further evaluation of the generic metacognitive model should include other types of distress and problems.

## Conclusions

In conclusion, the generic metacognitive model of common and frequently co-occurring distress symptoms provided a good fit to the data accounting for most of the variance in the latent distress construct. In the model, generic metacognitive beliefs and attentional control were correlated but accounted for unique variance. Our results support continued research on the generic model.

## Data Availability

The dataset generated and analyzed during the current study will be available in the NTNU Open Research Data respiratory once the manuscript has been accepted for publication. Until then, the dataset are available from the corresponding author on reasonable request.

## Abbreviations

ACS: Attentional Control Scale
CAS: Cognitive Attentional Syndrome
CBT: Cognitive-behavioral therapy
CFI: comparative fit index
FNE: Fear of Negative Evaluation
GAD-7: Generalized Anxiety Disorder Scale
IBM SPSS: International Business Machines Corporation- Statistical Package for the Social Sciences
MBSR: Mindfulness based stress reduction
MCQ-30: Metacognitions questionnaire 30
MCT: Metacognitive therapy
NICE: National Institute for Health and Care Excellence
NSD: Norwegian Centre for Research Data
PHQ-9: Patient Health Questionnaire
RMSEA: root mean square error of approximation
SEM: Structural equation modelling
S-REF: Self-Regulatory Executive Function
SRMR: standardized root mean square residual
TLI: Tucker Lewis index
